# Quantum mechanisms for selective detection in complex gas mixtures using conductive sensors

**DOI:** 10.1038/s41598-023-48207-0

**Published:** 2023-12-05

**Authors:** G. Kamarchuk, A. Pospelov, L. Kamarchuk, V. Belan, A. Herus, A. Savytskyi, V. Vakula, D. Harbuz, V. Gudimenko, E. Faulques

**Affiliations:** 1 B. Verkin Institute for Low Temperature Physics and Engineering, 47 Nauky Ave., Kharkiv, 61103 Ukraine; 2 National Technical University “Kharkiv Polytechnic Institute”, 2 Kyrpychov Str., Kharkiv, 61002 Ukraine; 3 SI “Institute for Children and Adolescents Health Care” of NAMS of Ukraine, 52-A Yuvileinyi Ave., Kharkiv, 61153 Ukraine; 4 Institut Des Matériaux Jean Rouxel (IMN), Université de Nantes, CNRS, F-44000 Nantes, France

**Keywords:** Biophysics, Gastroenterology, Health care, Medical research, Chemistry, Engineering, Nanoscience and technology, Physics

## Abstract

In this paper, we consider new quantum mechanisms for selective detection in complex gaseous media which provide the highest possible efficiency of quantum sensors and for the first time analyze their nature. On the basis of these quantum mechanisms, the concepts of quantum detection and innovative methods of analysis are developed, which are virtually impossible to implement in the conventional conductive sensors and nanosensors. Examples of original solutions to problems in the field of detection and analysis of human breath using point-contact sensors are considered. A new method of analysis based on detection of metastable quantum states of the "point-contact sensor—breath" system in dynamic mode is proposed. The conductance histogram of dendritic Yanson point contacts recorded for this system is a unique energy signature of breath which allows differentiation between the states of human body. We demonstrate that nanosized Yanson point contacts, which, thanks to their quantum properties, can replace a massive spectrometer, open up wide opportunities for solving complex problems in the field of breath analysis using a new generation of portable high-tech quantum sensor devices.

## Introduction

Ensuring sustainable progress in solving problems related to the analysis of complex gas mixtures requires the search for and application of universal detection mechanisms. As a result, the last few decades have seen a growing activity in the field of sensor studies. This is evidenced, for example, by one of the most promising types of chemical sensors based on the principle of electric conductance responding to the action of the analyzed substance^1–5^. The popularity of conductive sensors is due to the convenience with which they can be used to work with various types of objects, their high technological characteristics, the possibility of creating on their basis portable devices which can be integrated into existing devices, the low cost of the devices and their easiness to use. Their operation principles and mechanisms determine, to a large extent, the development trends in this area. One of the main approaches is based on the search for, selection, or synthesis of new materials, as well as their functionalization^6–9^. In a wide range of materials, much attention is attracted by polymers^10–12^, metal oxides^13–16^, various carbon materials such as carbon nanotubes and graphene^17–19^. Many of these are semiconductors, which improves the technological and metrological characteristics of the sensors. For example, the nature of semiconductors ensures the selectivity of sensors to donor or acceptor gases, depending on the semiconductor type.

In conventional sensors, the change in the electrical conductance of their sensing element caused by the action of the analyzed agent depends on the ratio of the thickness of the surface layer of the sample interacting with the external agent to the thickness of the entire sample^20^. In the general case, this leads to the fact that the sensitivity of a conventional sensor is largely determined by the geometric factor and depends on the ratio of the surface area of the sensing element to its volume. This predetermined the transition to miniaturization and use of nanostructured objects. Nanostructured sensing elements have additional advantages arising from the size effects, the possibility of creating a specific morphology of nanoparticles which form the sensitive layer of the sensor, the quantum confinement effects, etc. Among the most popular samples are nanowires, nanotubes, nanoclusters, nanoparticles of various geometry.

However, despite some successes, the traditional approach to the development of sensors within the framework of classical physics and chemistry has been exhausting itself gradually. Many developments have hit the ceiling of their capabilities and further progress is only observed in certain areas and certain parameters. This can be exemplified by the sensor detection in complex gaseous media. In the case of single-component gases, conventional sensors provide an acceptable level of reliability of results, but when we switch to multicomponent media, many problems arise and the reliability of conventional sensors turns out to be much lower, so careful control is needed for the results obtaining. This is particularly evident in the analysis of human breath, which is an ideal model object for solving detection problems in complex gas mixtures. Human breath, like any multicomponent medium, has a pronounced dynamic behaviour. It is characterized by the presence of a large number of interactions occurring between its components^21^. Human breath contains oxidizing agents (e.g., nitrogen oxides, carbon monoxide, sulphur oxide), reducing agents (e.g., hydrogen sulphide, ammonia, mercaptans, organic molecules) and other reactive elements. Over 2000 components of this medium contain highly reactive substances that can quickly interact with each other, creating the prerequisites for the emergence of new compounds. In turn, the newly formed substances can be catalysts for new chemical reactions, which means that progressive autocatalysis cannot be ruled out. This results in significant changes in the qualitative and quantitative composition of the system over a short period of time and can significantly affect the reproducibility of the results in the case of detection of a complex gas mixture by determining its individual components. This can lead to false positive or false negative results during diagnostic procedures^22,23^.

These problems can be avoided by switching to the use of new mechanisms of detection and new principles of analysis. One of them is the profiling approach that has become a very popular method in different areas. For example, registration of a spectral sensor profile of breath can provide the necessary information in real time and ensure high reproducibility of results^24–26^. However, extension of this approach to a wide range of conventional sensors has some fundamental limitations. The problem is that the manifestation of the characteristic features of the spectrum is due to quantum processes that involve transformation of energy. This means that appropriate tools are needed to register and analyze them. At the same time, conventional conductive sensors and the nanosensors that we are considering are hardly able to demonstrate a spectral character of the response because of the nature of the detection principles they are based on. The very nature of the way conventional sensors operate leads to limitations in their capabilities. As a result, conventional electrically conductive sensors cannot be used to register spectral processes and, accordingly, the spectral approach cannot be implemented on their basis.

Taking into account the operation mechanisms of conventional electrically conductive sensors, it is difficult to expect a variety of approaches to find solutions aimed at increasing their selectivity. Basically, the search for selectivity of conventional sensors is based on the principle of a key and a lock^27,28^. The development of sensor devices operating on the principle of a key and a lock is able to provide the creation of high-tech devices, but this approach also leads to limitations in the functional capabilities of the sensor and, as a result, to its narrow specialization. The old key will hardly be able to open a new lock. This makes it impossible to achieve universalization of the device needed to solve various problems and leads to the need for a significant change in the composition of the sensor material or modification of its design to allow detection of new objects.

A much higher efficiency of the sensor can be realized if its operation is focused not on individual components of the system, but on fundamental characteristics of the object under study, for example, on the energy parameters which characterize its nature. However, the energy parameters of an object, which determine its behaviour at the atomic level, also reflect its quantum nature, therefore, in order to register them, one needs a quantum instrument. Hence the obvious conclusion that one of the most promising approaches to achieving a breakthrough progress in the field of sensor development should be using "discrete" properties of materials. Quantum sensors can solve this problem.

## Materials and methods

### Peculiarities of the operation of quantum sensors at room temperature

Quantum sensors are brand new detectors based on fundamental quantum mechanisms and concepts. It is quantum mechanisms that provide the highest possible efficiency of such devices. The concepts of quantum detection lay the foundation for the development of innovative models that are virtually impossible to implement when creating conventional sensors and nanosensors. Let us consider some features of the operation of quantum sensing elements using electrically conductive sensors as an example.

With a decrease in the size of the nanostructures used as sensing elements of sensors, the nature of their behaviour changes, showing quantum limitations in the motion of charge carriers and the appearance of quantum size effects^29,30^. Quantum limitations manifest themselves when the size of the free electron localization area, for example, one of the characteristic dimensions of the sensing element of the sensor or particles of the sensor material, becomes comparable to the de Broglie wavelength

$$\lambda_{D} = \hbar /\left( {2mE} \right)^{1/2} ,$$

where *ħ* is the reduced Planck constant, *m* is the electron effective mass, and *E* is the electron energy. For semiconductors, the Bohr radius of the exciton *r*_n_ = *m*_0_*ε*_r_*a*_0_*n*^2^ / μ serves as such a criterion. Here, *m*_0_ is the electron mass, *ε*_r_ is the relative permittivity of the medium, *n* is the exciton quantum number, *μ* is the reduced electron mass, and *a*_0_ is Bohr radius: *a*_0_ = (4π*ε*_0_ℏ^2^) / (*e*^2^*m*), where *ε*_0_ is the permittivity of free space, and *e* is the electron charge. As a rule, the de Broglie wavelength in metals is in the range of 0.1–10 nm, while the Bohr exciton radius in semiconductors is 1–10 nm.

In nanostructures, the free movement of electrons can be limited to one, two, or three directions. With a decrease in the characteristic dimensions of a nanoobject, the exciton binding energy increases, and, as a result, quantum limitations can manifest themselves even at room temperature. Due to the quantum limitations, electrons placed in a limited space can only occupy discrete energy levels. The restriction of the free movement of electrons and the discreteness of their energy states, in contrast to the case of free movement in bulk objects, leads to a difference in the properties of nanostructured sensing elements and those of their bulk counterparts. For example, nanostructured sensors exhibit chemical activity which is significantly different from that of bulk objects^31^. In nanocrystalline materials, the charge transfer rate is mainly determined by the effects of quantum confinement and differs significantly from that in classical homogeneous samples characterized by the free motion of electrons. As a result, non-traditional detection mechanisms may appear in the case of nanostructured sensing elements. This makes it possible to develop new principles and open new areas of operation of sensor devices.

One of these areas is the approach based on the energy principles of detection and analysis^25^. A quantum system has a set of metastable energy states which form its energy profile (signature) and uniquely characterize it. This means that knowledge of the energy profile of a system provides unlimited possibilities for its recognition and analysis. This leads us to a simple conclusion: for effective detection and analysis of a system, it is sufficient to determine the energies of its metastable quantum states^32^. It may seem that researchers have now an instrument for developing a universal tool which is capable of selectively detecting any quantum object. However, this is not that simple. At room temperature, competing phenomena come into play that mask the manifestation of quantum effects, and creation of quantum tools is considerably more complicated. For example, observation of quantum effects in electrical conductors at room temperature becomes very problematic because of the temperature broadening of the energetic levels, including the Fermi level, in the energy spectrum of electrons and the competing influence of thermally induced processes blurring the object's quantum nature. This leads to the fact that at room temperature electrically conductive materials, known to be among the most promising objects for creating chemical sensors and nanosensors and in most cases used conventionally, become unsuitable for the implementation of quantum detection mechanisms and creation of quantum sensors. In addition, in conventional sensors, in contrast to the requirement to remove thermal effects which can hinder the manifestation of quantum processes, a frequently used approach is to heat the sensing element in order to increase the intensity of the response and accelerate the desorption of atoms from the surface of the sensing element^33^. For example, operation of sensors based on metal oxides requires high temperatures because of the material's large bandgap. At the same time, an attempt to cool a semiconductor sensor in order to reduce the contribution of thermal fluctuations inevitably leads to its transition to the insulator state.

To implement the energy concept of quantum detection, it is necessary to have a tool for determining the energy of specific states of the objects under study. In the case of sensors used to detect atomic and molecular substances, such information can be accumulated by measuring the energy of adsorption of atoms and molecules on the sensor surface or by determining the interaction energy of the systems of quasiparticle excitations that characterize the sensor material and analyzed object. This can be seen most clearly in the example of sensors with the operation principle of changing electrical conductance.

The properties of a traditional electrical conductor are largely characterized by the features of the two main systems of quasiparticles—electrons and phonons. These systems of quasiparticles have quantum properties. The system of electrons determines the electrical conductivity of the conductor. Phonons are a system of quasiparticles which describes the vibrations of the crystal lattice of the material^34^. Quantum systems of electrons and phonons in a metal can serve as a tool to selectively detect various external agents^20,25,35^. The interaction of electrons and phonons with each other or with the atoms and molecules adsorbed on the surface of the sensor's sensing element makes it possible to obtain all necessary energy information about the sensor material, as well as the data needed to identify the analyzed agent^25^. The energy approach is a universal principle that ensures the absolute selectivity of quantum sensors^32^. This looks very promising from the viewpoint of creation of innovative devices. However, implementation of this principle at room temperature encounters certain difficulties. One of the main obstacles in this case, as has already been mentioned, is the thermal effects. To avoid their influence, quantum properties of materials are usually studied at low temperature^36^.

We can consider the following fundamental reasons which result from the thermal effects and affect the possibility of obtaining information about quantum processes at room temperature. The first is related to the influence of thermal effects on the electron distribution function in a metal. At non-zero temperatures, there is a thermal broadening of the Fermi level, which characterizes the energy of electrons that can participate in the conduction of the metal at a given temperature. It is related to the thermal excitation of electrons located at energy levels near the Fermi level. This effect has a great influence on the solution of the problem of direct registration of spectral interactions. For example, in the case of direct measurement of the spectra of quasiparticle interactions, in particular, those of the interaction between electrons and phonons, an increase in temperature results in the narrow delta-type spectral lines turning into broad bell-shaped bands^29^. This affects the resolution of the spectral instrument and leads to the fact that observation of the spectral bands with a width smaller than the thermal broadening of the spectral lines becomes impossible.

The second reason is related to the dominance of thermal effects at room temperature, which can hide any non-linear non-equilibrium processes in materials and lead to the suppression of the quantum phenomena. One of the options for eliminating this negative influence can be spatial separation of the non-equilibrium processes, which characterize the quasi-particle interactions occurring with a change in energy, and the thermal ones.

To make electrons serve as a probe for the energy of phonons, which characterize the vibrations of atoms of the crystal lattice or the normal vibrations of the atoms adsorbed on the surface of the detector, it is necessary to make the electron system nonequilibrium at the energy level of the corresponding vibrations without letting the effects of crystal lattice heating manifest themselves. In other words, electrons must have some excess energy which they could transfer to phonons and thus change their energy state. For this to happen, the nonequilibrium energy processes resulting from this interaction and the thermal processes, which cause chaotic oscillations of atoms and disrupt the interaction between the quasiparticle systems in their pure form, must be separated in space. In a homogeneous conductor, this can hardly be achieved even at low temperatures. But in an inhomogeneous conductive system, spatial separation of thermal and nonequilibrium processes can be successfully obtained. The Yanson point contact is an example of such a system^34,37^. The essential difference between a Yanson point contact and a homogeneous conductor is the possibility of separating in space the thermal and nonlinear electric current phenomena associated with the nonequilibrium state of the electron gas^38^.

A Yanson point contact is an electrical contact created over a small area between two bulk metal electrodes which meets a set of specific criteria of the Yanson point-contact spectroscopy^36^. One of the criteria is the fulfillment of the inequality *d* < < *l*, where *d* is the contact diameter and *l* is the mean free path of electrons in the contact material. This criterion defines the Yanson point contact as a nanostructure with a size ranging from the size of one atom to several tens of nanometers. Unlike traditional electrical contacts, the Yanson point contact has a number of unique physical properties, which include the possibility of observing spectral interactions with various types of quasiparticles^34,39^, the quantum nature of electrical conductance^40^, the point-contact gas-sensitive effect^20,35^, the cyclic switchover effect^41^, etc.

Hypothetically, in the case of a homogeneous conductor, even if we could obtain electrons with excess energies sufficient to excite lattice vibrations, it would be virtually impossible to implement the observation of a nonlinear current–voltage (*I*-*V*) characteristics of a homogeneous sample, since already at the initial stage of recording the *I*-*V* characteristics destruction (melting, sublimation) of the material of the object under study would occur. This is due to the fact that in a homogeneous conductor the processes of inelastic scattering of electrons, which contribute to the electrical resistance of the conductor, and the processes of energy relaxation are not spatially separated and occur uniformly throughout the entire volume of the sample. In practice, a homogeneous sample has enough time to melt before the electron gas can acquire an excess energy comparable to the energy of the lattice vibrations. Indeed, in order for electrons to acquire an excess energy in an electric field which is comparable to the characteristic energies of phonons *ħω*_D_, it is necessary to provide a current density of the order of 10^9^ A/cm^2^, which follows from the relation *eEl* ~ *ħω*_D_^36^. Here *ħ* is the reduced Planck constant, *ω*_D_ is the Debye frequency of phonons, *e* is the electron charge, *E* is the electric field strength, and *l* is the mean free path of electrons in the metal. Metal melting occurs already at current densities of the order of 10^2^–10^3^ A/cm^2^, i.e., the average excess energies of electrons that can be achieved in practice in the homogeneous case are very small compared to the characteristic energies of phonons. Thus, thermal phenomena are a serious obstacle to the realization of quantum effects.

To avoid the influence of thermal effects, it is necessary first of all to make sure the crystalline structure of the sample is homogeneous and defect-free. This makes it possible to achieve large mean free paths of charge carriers in conductors. Defect-free and homogeneous crystalline structures can be achieved in highly perfect single crystals. Film structures, which are very often used to create sensors, do not meet these criteria because of the technology of their manufacture. The mean free path of electrons in such objects is much shorter than that in single-crystal samples.

The mean free path of electrons in a metal is a limiting factor for observing quantum effects. It determines the possibility of realizing the ballistic mode of electric current flow in the conductor, which in some cases makes it possible to avoid the influence of thermal effects. Ideally, the longer the mean free path of charge carriers, the easier the separation of the thermal phenomena and current phenomena related to the nonequilibrium electron gas. The Yanson point contact allows us to achieve this in a small nanoscale region of space because of the inhomogeneity of its structure and unique physical properties. As we have already mentioned, the Yanson point contact is a nanoobject with characteristic dimensions ranging from the size of one atom to several tens of nanometers^37^. At low temperatures, for example, at 4.2 K (liquid helium temperature), the mean free path of electrons in metals can reach a few micrometers or more. In this case, the diameter *d* of the Yanson point contact fulfils well the relation *d* < < *l*, at which the ballistic current flow mode is realized^38^. As a result, a favourable situation is created in the ballistic regime, when spectral quantum phenomena of interaction between electrons and phonons can occur in pure form in a small region of the material. Electrons scatter on phonons, transfer to them their excess energy acquired in the electric field of the Yanson point contact, and excite vibrations of atoms of the contact material. In this case, thanks to the long free path of phonons, the thermal energy is carried far beyond the contact and absorbed by the massive "banks"—the electrodes—which ensures that the contact material does not heat up. As a result, the quantum phenomena of electron–phonon interaction can be easily detected, thus providing the implementation of the Yanson point-contact spectroscopy^34,42^.

At room temperature, it is much more difficult to implement the ballistic mode of current flow, since in this case the mean free path of electrons is significantly reduced compared to that at low temperatures. However, the special technology proposed in Ref.^41^ allows one to create, for example, a dendritic Yanson point contact with a perfect crystalline structure, in which the maximum electron mean free path attainable for the given conditions can be achieved. According to the estimates of Ref.^43^, the mean free path of electrons in pure copper reaches 300 Å at room temperature. This means that in dendritic Yanson point contacts, which have a perfect crystalline structure, we can obtain a ballistic regime of electric current flow in the resistance range of 10 Ohm–12.9 kOhm. Electrochemical synthesis of the material, occurring during the creation of dendritic Yanson point contacts, provides a perfect crystalline structure and a defect-free material in the contact area. This allows separation of the nonlinear current phenomena and the thermal effects in the contact, transferring the manifestation of the latter to a space far beyond the contact. As a result, the Yanson point contact can be used as a sensing element of a quantum sensor that operates effectively at room temperature^25,26,32^. At the same time, it should be noted that the use of the repeatedly proven technology of the Yanson point contact spectroscopy ensures the creation of point contacts with well-reproducible properties. This makes it possible to easily fabricate nanostructured point-contact sensors in which quantum confinement effects are observed^32,41^.

When operating in the regime of registration of the spectral profile of a complex gas mixture, point-contact sensors can help us to avoid the above-mentioned problem of reduced information content of the measured dependencies caused by the thermal broadening of the spectral lines. The point-contact sensor profile of breath is not a quasi-particle interaction spectrum in its pure form. It is a spectrum-like temporal dependence of the electrical conductance of the sensor, which reflects the energy processes of interaction occurring in the quantum system under study. When measuring the spectral sensor profile of breath, we register not the spectral line itself, but the result of a change in the resistance of the Yanson point contact at a certain energy which corresponds to the adsorption energy of the gas atoms and molecules. The change in the electrical conductance of the Yanson point contact is due to the quantum process of transferring the excess energy of nonequilibrium electrons to atoms adsorbed on the surface of the point contact and their desorption. It is the fact of desorption of an atom or molecule from the surface of the point contact, taking place when a certain value of the transferred excess electron energy is reached, which is recorded as a feature in the dependence *R*(*t*) of the contact resistance *R* on time *t*. And since the thermal processes are brought outside the Yanson point contact, they do not affect the registration process, do not contribute to the measured data, and thus do not reduce the information content of the spectral profile of human breath.

### Samples fabrication

The methodology of quantum Yanson point contacts creation utilized for point-contact sensors is described in detail elsewhere, see for example, Ref.^37^. The basics of designing various types of Yanson point contacts have been developed within the framework of the Yanson point-contact spectroscopy technology^36^. The most commonly used methods for creating point-contact sensing elements are the "needle-anvil" method, the Chubov displacement technique, and the "break junction" method^20,35,37^. The technology for creating point-contact sensors for studying human breath is described in detail in Refs.^24,25,44,45^. The main elements of the process of creating dendritic Yanson point contacts are presented in Refs.^32,41,46^. All procedures and methods were performed in accordance with the relevant guidelines and regulations.

### Data registration

Measurements of the electrical parameters of point-contact sensors were carried out using standard measurement equipment, including a lock-in amplifier (SR 830 DSP) and various types of Keithley multimeters. When it was necessary to measure small signals at the nanovolt level, an original point-contact spectrometer developed at B. Verkin Institute for Low Temperature Physics and Engineering^47^ was used. To analyze human breath with quantum point-contact sensors, an original portable device developed by the authors of this work was used. Details on the procedure for conducting point-contact breath tests can be found at http://qs.net.ua/results_eng.html.

### The cyclic switchover effect

Dendritic Yanson point contacts were formed in the needle-anvil geometry. Copper electrodes were placed in a special device described in Refs.^32,46^. Pure water was used as an electrolyte. The methodology for obtaining and studying the cyclic switchover effect is presented in detail in Refs.^32,41,46^. The procedure for constructing conductance histograms is described in detail in Refs.^32,41,48^.

The research protocol was approved by the Institutional Review Board and the Ethical Committee of the SI “Institute for Children and Adolescents Health Care” of the NAMS of Ukraine. All volunteers gave their written consent to participate in the study.

## The problem analysis

### Spectral quantum mechanisms of selective detection in complex gas mixtures

In the next sections, we consider for the first time the nature of the quantum mechanisms for detection in complex gas mixtures using conductive sensors. The analysis has allowed for necessary generalizations that serve to demonstrate the universality of the considered detection mechanisms. Universality of the mechanisms is understood in a broad sense. It is not only the ability to identify the composition of various objects and media, but also the ability to develop numerous detection methods based on it. To demonstrate this, we have considered the results of developing methods for detection in complex gaseous media using the energy of interaction of quasiparticles, the approaches based on a single characteristic parameter, as well as using a set of characteristic parameters of the spectral sensor profile of a complex gas mixture. The universality of the quantum detection mechanisms provides high reliability and accuracy of results by means of using several detection methods developed on its basis.

Quantum point-contact sensors provide ample opportunities to formulate and implement various concepts for the detection and analysis of human breath on the basis of the spectral quantum mechanism of selective detection in complex gas mixtures. At present, several detection concepts have already been proposed and tested, which can be implemented using point-contact sensors^24–26^. A set of diagnostic methods based on the principles of breath analysis performed with quantum point-contact sensors can provide a comprehensive solution to a number of diagnostic problems within a single technology. In this and the following section, we consider for the first time a new concept for the development of advanced diagnostic methods using the quantum mechanism for selective detection with point-contact sensors in gaseous and liquid media based on conductance quantization^32^ and briefly describe the methods which have already been tested in medical practice. It should be noted that a distinctive feature of the presented methods is the execution of all procedures in real time.

### The concept of spectral analysis of the sensor profile of breath

The spectral nature of the response of quantum point-contact sensors to the action of a complex gas mixture makes it possible to implement the concept of spectral analysis of the sensor profile of breath. The spectral response of quantum point-contact sensors^37,49^ reflects the fundamental properties of Yanson point contacts comprehensively studied in the Yanson point-contact spectroscopy. The principle of operation of a quantum point-contact sensor in the spectral mode of recording a response signal can be illustrated by analysis of a typical spectrum of electron–phonon interaction in a metal recorded during spectral studies^34,36^. For simplicity's sake, we represent the Yanson point contact in the model of a long conductive nanochannel connecting massive current-feeding electrodes^37^. One of the unique properties of this nanoobject is the specific distribution of the potential when an electric current flows through its conduction channel^38,50^. In the Yanson point contact, which can be represented as an "electrode—point contact—electrode" system, the entire potential drop caused by the flow of electric current occurs in the area of the point contact. At the same time, virtually no potential drop is observed on the current-feeding electrodes. As a result, the potential difference is formed only on the conduction channel of the Yanson point contact. This means that the entire resistance of the "electrode—point contact—electrode" system is determined by the Yanson point contact, which is responsible for the change in the resistance of the entire system when interacting with a gaseous medium^20,35^.

**Figure 1:**
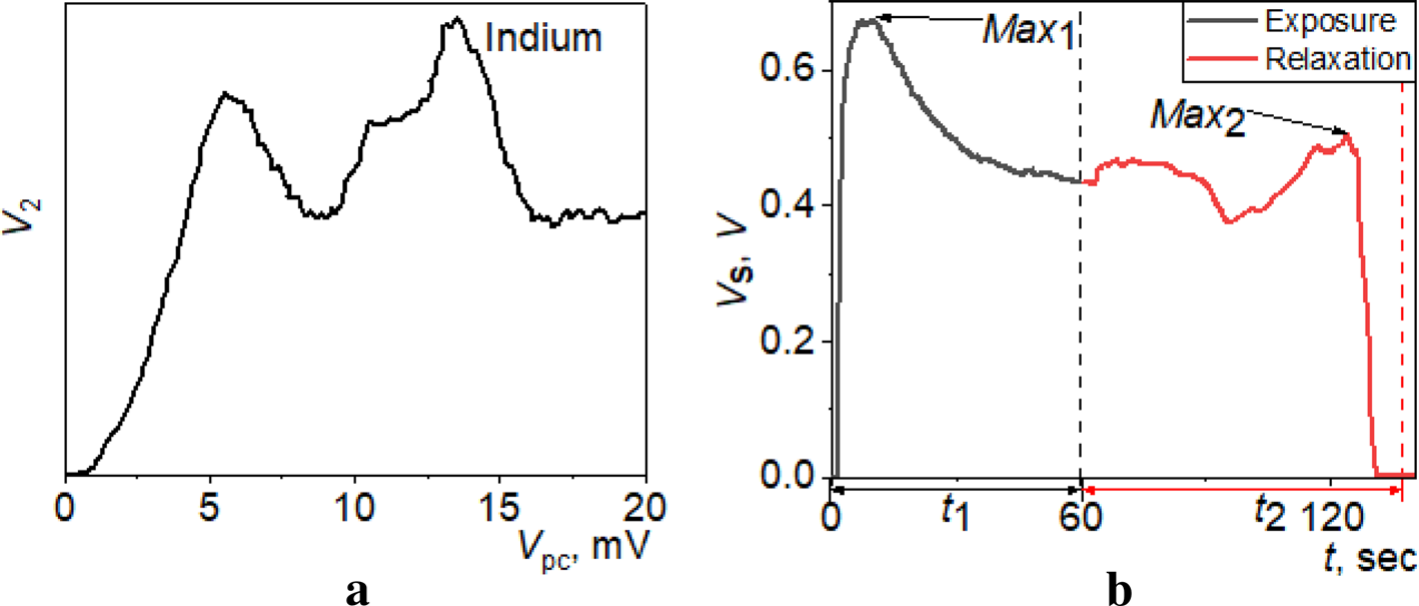
Spectral dependencies recorded using Yanson point contacts. (**a**) spectrum of electron–phonon interaction in indium; (**b**) spectral profile of breath. *V*_2_ is the second derivative of the current–voltage characteristics of the point contact; *V*_pc_ is the voltage drop across the contact; *V*_s_ is the voltage drop across the sensor; *t* is the time; *t*_1_ is the sensor exposure time; *t*_2_ is the sensor relaxation time. Data are taken from Ref.^25^.

Thanks to this peculiar potential drop, the entire electric field is concentrated in the area of the conduction channel of the Yanson point contact. As a result, as electric current flows, electrons moving through the conduction channel are accelerated by this electric field and acquire an excess energy with a value of the order of *eV*, where *V* is the voltage applied to the contact. This process can be modeled by a special electron momentum distribution function. The interaction of electrons with the lattice sites and the subsequent relaxation of this function are reflected in the current–voltage characteristics of the contact in the form of characteristic nonlinearities^38^. An electron which has acquired some excess energy in the contact in the process of current flow interacts with an atom of the crystal lattice, excites a phonon with an energy *ħω* = *eV* by transferring to it its excess energy, and relaxes down to the ground state. Because of the long mean free path of electrons and phonons in the contact, all the thermal energy in this process is carried far beyond the contact and absorbed by the bulk electrodes. This is how the non-equilibrium current and thermal processes are separated in space. If we carry out a sweep of the electric current in the contact, which provides a change in the voltage on the contact in the range from zero to *eV*_max_ = *ħω*_D_, where *ω*_D_ is the Debye frequency of phonons, then the process of scattering of electrons by phonons can be directly recorded in the form of the second derivative d^2^*I*/d*V*^2^(*V*) of the *I*–*V* characteristics, which is called the point-contact spectrum^34,36^. This dependence directly contains complete information about the function of density of phonon states in the contact material, that is, it demonstrates the energy distribution of phonons from zero to *ħω*_D_. An example of point-contact spectrum in indium measured at the temperature of liquid helium is shown in Fig. 1a.

A breath profile recorded at room temperature using a point-contact sensor (Fig. 1b) reflects similar quantum processes, but in this case, there is an interaction between nonequilibrium electrons and molecules of the gaseous medium adsorbed on the surface of the conduction channel of the Yanson point contact. This interaction will be effective and capable of causing a noticeable change in the electrical conductance of a point contact if the excess energy of electrons is comparable to the energy of adsorption of the gas molecules. In the Yanson point contact, this situation becomes real thanks to the specific potential distribution. It is precisely because of this distribution that, when electric current flows in the contact, electrons can be accelerated in the electric field of the contact and acquire an energy which is comparable to the adsorption energies of the gas molecules on the contact surface. Observation and registration of the processes of interaction of conduction electrons with the gas molecules adsorbed on the surface of the conduction channel become possible thanks to another original property of Yanson point contacts mentioned above. This property consists in the spatial separation of nonequilibrium processes of interaction of quasiparticle excitations in the contact and heat release processes by means of moving the latter far beyond the Yanson point contact. This possibility is ensured by the long mean free path of quasiparticles in the contact and removal of the thermal energy of the gas molecules from the contact area after their desorption.

The adsorption of gas molecules on the surface of a Yanson point contact creates additional scattering centers in the contact material. The appearance of defects in the crystalline structure leads to a reduction in the mean free path of electrons and increases the time electrons spend in a given area of the contact. This increases the probability of electron scattering at the sites of the adsorbed molecules. As a result, the number of cases of interaction between electrons and the gas molecules increases with the transfer to the latter of the excess energy acquired by electrons in the electric field of the contact in the process of electric current flow. The molecules, upon receiving an energy comparable to the energy of adsorption, desorb from the surface of the conduction channel of the Yanson point contact. It should be noted that the ultra-high current density of the order of 10^7^ A/cm^2^, which can be achieved in the Yanson point contact even at room temperature without any negative consequences for the mechanical stability of the sensor^20^, provides a guaranteed transfer of the energy necessary for the molecules to desorb. The desorption of molecules from the surface actually reflects the fact of scattering of charge carriers in the contact leading to a change in its electrical conductance, which manifests itself in the dependence of the contact resistance *R* on time *t*. The process occurs in real time. The duration of the interaction reflects the amount of the energy transferred. Since different gas molecules have specific adsorption energies that are characteristic of them, adsorption of different molecules can be separated in time and recorded in different parts of the *R*(*t*) curve.

This becomes possible thanks to the ultra-high sensitivity of the point-contact sensor to changes in its electrical conductance. The Yanson point contact allows reliably recording changes in its electrical conductance at the level of one conductance quantum. The interaction of a Yanson quantum point contact with a gas molecule can lead to a change in the contact resistance by 12.9/*n* kΩ, where *n* is the number of atoms in the contact^32,41^. For a single-atom contact corresponding to one conductance quantum, this value is 12.9 kΩ. Such a big change in electrical resistance can easily be recorded using conventional electrical measuring instruments. This makes it possible to record with high resolution all processes of interaction with breath molecules and obtain a highly informative energy profile of this gas mixture. Thus, similarly to the point-contact spectra of electron–phonon interaction, the point-contact sensor profile of breath contains information about the energy parameters of the atoms and molecules adsorbed on the surface of the Yanson point contact. In contrast to conventional sensors utilizing the principle of changing electrical conductance, thermal processes in Yanson point contacts do not affect the interaction between electrons and adsorbed molecules and do not hide the result of this interaction.

To decipher the spectral profile of breath obtained using point-contact sensors, both the various spectral analysis techniques already tested in the Yanson point-contact spectroscopy can be used^34,36^ and new approaches to extraction of the necessary information can be developed^25,51^. The idea implemented in Ref.^25^ is that in order to detect a component of a complex gas mixture, it is necessary to determine the spectral profile section which corresponds to the energy of the interaction of electrons in the Yanson point contact with this specific component. To determine the section of the spectral breath profile which is responsible for the interaction with this component of the analyzed environment, we can apply the following approach. It is necessary to record the breath profile of a patient with a point-contact sensor and determine the content of the component, selected as a control substance, in the patient's body using a standard and well-established method. Then we need to conduct these measurements for a certain number of people in order to accumulate a sufficient amount of data for statistical calculations. Having the necessary set of data, we can process it statistically and find the dependence of the correlation coefficient of the studied component of the gas medium on the sensor response signal during the analytical procedure. After that, we can select a section of the maximum correlation in the spectral breath profile, determine the average value of the response signal in this section, and, using the entire data set, find the dependence of this value on the concentration of the studied component. In this way it is possible to obtain a regression equation which can be used as a calibration characteristic to determine the concentration of the desired component of a complex gas mixture.

This approach was developed and successfully tested by finding concentrations of such important hormones of the human body as serotonin, cortisol, and melatonin^25,52^. This allowed us to offer simple equations to determine the concentration of those hormones in real time by recording point-contact profiles of breath. For example, the equation for determining serotonin concentration had the following form:

$$C_{{{\text{ser}}}} [\upmu {\text{mol}}/{\text{l}}] = \, 1.17 \, {-} \, 2.41 \times \overline{V}_{s} ,$$

where ${\overline{V}}_{s}$ is the value of the average voltage of the metabolic profile of a particular patient for the period of the greatest correlation with the hormone concentration in the human blood. The concentration of cortisol and melatonin in the body was determined just as simply. As a result, by recording a spectral profile of breath and measuring the voltage drop on the point-contact sensor, we can immediately obtain the necessary diagnostic information about the concentration of hormones in the human body in a noninvasive way. Implementation of the principle of spectral analysis of sensor profiles of breath by using the obtained regression equations creates the basis for the comprehensive development of quantum sensors and new diagnostic methods for monitoring the hormonal state of the human body.

### Concept of characteristic parameters of the quantum sensor response curve for analysis of the human breath spectral profile

Point contact sensors visualize the spectral profile of breath by providing a time sweep of the entire energy interactions spectrum in the quantum system "point contact—breath". The spectral profile of the breath, like other spectral dependencies, can be described with a large number of parameters that characterize various quantum processes that take place during its registration. These characteristic parameters contain information about both the absolute values of specific quantities and generalized information about particular processes. To demonstrate the effectiveness of this concept, let us consider some of the numerous characteristic parameters of the point-contact sensor response curve^45,53^.

**Figure 2:**
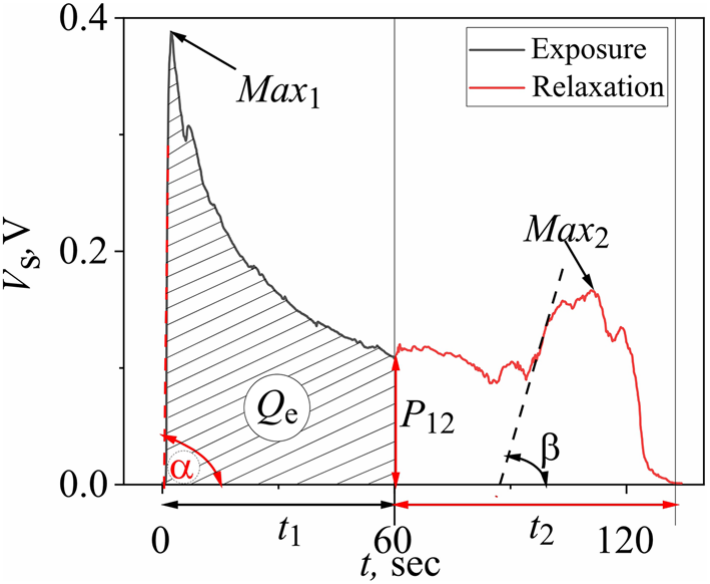
Spectroscopic breath profile obtained by the point-contact sensor based on the TCNQ compound. Data are taken from^26^. *V*_s_—voltage drop that occurs in the sensor; *t*—response time; characteristic parameters of the response curve: *t*_1_—exposure time; *t*_2_—relaxation time; *Max*_1_—exposure maximum; *Max*_2_—relaxation maximum; *p*_12_—ordinate of the final segment of the exposure phase; *d*_1_ = tg*α*—slope of the initial section of the exposure maximum; *d*_21_ = tg*β*—slope of the initial section of the most intensive maximum in the relaxation period; *Q*_e_—area under the exposure curve.

Each peculiarity in the breath spectral profile, such as maxima, minima, areas of monotonic or non-monotonous changes, etc., occurs at a certain stage of the registration process and, therefore, reflects the corresponding aspects of the interaction of the sensor sensing element with the gas medium in a certain period of time. Effects caused by quantum interactions can be observed in their pure form due to the absence of thermal factors influencing the nonlinear electrical conduction in the Yanson point contact. For example, the absolute value of the point-contact sensor response signal in a maximum makes it possible to draw a conclusion about the interaction intensity. The concentration of the breath components and their adsorption energy affect the dynamics of the interaction processes with the sensor material. The behaviour of the spectral profile in the initial section of the curve is mainly determined by the adsorption processes involving the most chemically active components of the breath. As a result, a variation in the composition and concentration of the breath components for corresponding persons leads to a change in the response signal rise rate which, together with other parameters, provides the possibility of distinguishing one gas mixture from another. The parameter which reflects the processes occurring in the first part of the exposure period is the slope of the initial section of the maximum in the exposure period *d*_1_ = tg*α* (Fig. 2).

The opposite process, associated with the desorption of atoms or molecules after their interaction with sensor material, proceeds under conditions different from those at the initial stage of registration. Therefore, an asymmetric behavior of the response curve during the exposure and relaxation is observed. As a result, the signal rise rates in the areas of exposure and relaxation maxima are different as well. The interaction efficiency of the nonequilibrium electron flow in a Yanson point contact with the gas molecules located on the surface of a point contact depends on the nature of the molecules absorbed. Different gases absorbed on the sensor surface cause different distortions of the crystalline structure of the Yanson point contact conduction channel. As a result, the change in the electron mean free path in the vicinity of the sites of adsorbed molecules and the interaction times of nonequilibrium electrons with the adsorbed molecules differ in each specific case. In turn, the change in the electrical conductance of the Yanson point contact also depends on the donor or acceptor nature of the adsorbed gas molecules. As a result, variations in the time and energy of the interaction of electrons with atoms or molecules of various adsorbed substances cause a change in the rise rate of the quantum sensor response signal in a given temporal section of the relaxation curve, which characterizes the specific component and the composition of the breath as a whole. The parameter which reflects the processes occurring in the first part of the relaxation period is the slope of the initial section of the most intensive maximum in the relaxation period *d*_21_ = tg*β* (Fig. 2).

Another important characteristic parameter of the spectral profile of human breath is the area under the exposure curve *Q*_e_. This is an integrated characteristic of all interactions of nonequilibrium conduction electrons in the Yanson point contact with a complete set of the analyzed gas medium components. All interactions that provide a significant contribution to the contact conductance change during the adsorption of atoms or molecules on the surface of the Yanson point contact conduction channel can be described by this parameter. And, since the sensitivity of a point-contact sensor to the conductance change is at the level of one conductance quantum, the contribution of any atom or molecule adsorbed on the sensor surface is unambiguously taken into account by this characteristic parameter.

The value of the sensor electrical conductance at the end of the exposure period is determined by the ordinate of the final section of the exposure curve *p*_12_ (Fig. 2). The sensor response signal in this area is formed when the process of adsorption of breath components on the surface of the point-contact sensor ends and the gas mixture on and near the surface of the point contact transfers to a new quasi-equilibrium state. The components of a metastable structure consisting of the adsorbed molecules of the gas mixture can make the opposite contributions to the contact conductance. For example, donor and acceptor gases lead to processes of different direction with respect to the charge transfer and, accordingly, the predominance of one or the other process leads to an increase or decrease in the electrical conductance of the Yanson point contact^35^. The cumulative result of all interactions on the surface of the point-contact sensor determines the final electrical conductance of the quantum system "point-contact sensor—human breath". The integral characteristic of this process is the parameter *p*_12._

A combination of the various characteristic parameters of the spectral breath profile is able to display the most characteristic features of the interaction of the conduction electrons in the Yanson point contact with the analyte and, thus, identify the latter among many other components. Two approaches are currently used within the concept of characteristic parameters. The first approach is to search for a correlation between the certain characteristic parameter of the spectral sensory breath profile and the gas agent under study or the state of the analyzed medium. For example, the integrated value of the absorption energy of the patient breath components determines the duration of the point-contact sensory breath profile in the relaxation phase. As a result, the time scale during the sensor relaxation is a reflection of all energy desorption processes in the “point-contact sensor—human breath” system, which makes it possible to identify the object under study.

Due to its energy nature, the sensor relaxation time *t*_2_ (Fig. 2) corresponds to a certain breath profile and thus allows distinguishing between the metabolic processes of different patients. In fact, similar to the electron–phonon interaction spectra in the Yanson point-contact spectroscopy, the point-contact spectral profile of a gas mixture of certain composition can be characterized by its particular length^26^. Since the relaxation time is a quantity that reflects the integrated characteristic of the energy of all interactions of a point-contact sensor structure with the analyzed medium, the length in time of the spectral profile during the relaxation period has a particular value for each gas mixture corresponding to particular conditions. Based on this, the sensor relaxation time *t*_2_ characterizes in an integrated manner the energy nature of the object and can be used to identify it. In this case, there is no need to determine the individual components of the quantum system for making a diagnosis.

An example of using the characteristic parameter *t*_2_ for diagnostic purposes is the detection of carcinogenic strains of the *Helicobacter pylori* bacterium^24^. The World Health Organization recognizes *Helicobacter pylori* infection as the primary causing factor in development of peptic ulcer disease, gastric cancer, and gastric mucosa-associated lymphoid tissue (MALT) lymphoma^54^. It is the toxigenic strains of *Helicobacter pylori* which are responsible for appearance of the above diseases; while non-toxigenic strains cause a disease in a minimum number of cases^55^. In Ref.^24^, a correlation was found between the relaxation time of the quantum point-contact sensor response signal and the breath of patients who had certain body conditions associated with the presence of this infection. As expected from the physical meaning of the characteristic parameter *t*_2_, the relaxation time of quantum point-contact sensors was well reproduced when recording the breath spectral profile, which was specific for certain patient conditions. The presence of carcinogenic strains of the *Helicobacter pylori* bacterium in the human body led to a significant change in the length of the spectral breath profile due to an increase in the relaxation time. This made it possible to offer the first breath test which could detect carcinogenic strains of the *Helicobacter pylori* bacterium in real time. This example demonstrated the high efficiency of quantum point-contact sensors and of the concept of using characteristic parameters to analyze the spectral profile of breath.

The second approach based on this concept is mathematical modeling of the analyzed object and description of its state using a regression equation that includes several characteristic parameters of the spectral sensory breath profile. This approach was tested in experiments on characterization of the hormonal state of the human body.

The characteristic parameters provide a tool for quantitative assessment of the features in the point-contact sensor response curves. They demonstrate certain relationship with each other and are directly related to the physical and chemical processes in the "point-contact sensor—human breath" system. This means that their combinations can unambiguously characterize the state of a complex gas mixture and its components. Having both data of measurements for certain substances characterizing human metabolic processes and spectral sensory profiles of human breath, it is possible to use the described approach and check its efficiency. A possibility of solving this problem for such important hormones of the human body as serotonin and melatonin was demonstrated in the framework of experimental data regression analysis^26^. As a result, a simple relationship was proposed, which made it possible to determine the concentration of serotonin in the patient's blood and rank patients into groups by certain hormone concentration ranges:

$$C_{ser} \left[ {\frac{{\upmu {\text{mol}}}}{{\text{l}}}} \right] = 5.2369 - 0.9040 \times t_{2} - 4.1749 \times p_{12} - 4.4346 \times Q_{e} .$$

This equation serves as a calibration dependence for sensory determination of the concentration of a particular component of a complex gas medium using a point-contact breath test. The observed strong negative linear correlation between the concentration of serotonin in the patient's blood and the characteristic parameters of the point-contact sensory breath profile led to the conclusion that the measurement of the sensory breath profile allows assigning patients to one of the groups in the range of serotonin concentration 0.1–1.2 µmol/l in real-time mode. A similar regression equation was obtained to determine in real time the concentration of melatonin in the urine. In this case, the measurement of the sensory breath profile made it possible to assign patients with high accuracy to one of the groups in the melatonin concentration range of 10–130 nmol/l.

Thus, the characteristic parameters of the spectral breath profile contain comprehensive information for the analysis of certain components of this complex gas mixture. It makes it possible to implement a non-invasive test for monitoring the hormonal state of the human body. The reliability of this information can be guaranteed by using a combination of two concepts for the analysis of complex gas mixtures based on the data obtained with quantum point-contact sensors: the concept of spectral analysis of the sensor breath profile and the concept of characteristic parameters. As we can see, breath tests based on quantum mechanisms of selective detection in complex gas mixtures make it easy to monitor the dynamics of changes in human hormonal background and find concentrations of serotonin, cortisol, and melatonin in the human body in real time. The proposed approaches to analyzing breath using quantum point-contact sensors provide unlimited possibilities for the development of a wide range of methods of medical diagnostics.

## Results and discussion

### Conductance quantization as a new mechanism for selective detection in human breath

A quantum physical system is characterized by a set of energy eigenstates which corresponds to the local minimum of the total system energy. A specific set of such states is only characteristic of a certain system, being its individual energy “signature” at the atomic level. Knowledge of the energy parameters of the signature is a key factor for determining and predicting the properties of the physical object. This quantum tool to determine the metastable states of a system can become the basis for the development of quantum sensor technologies. The mechanism for recognizing substances by determining their energy parameters provides brand new possibilities of differential analysis of various states of physical objects and makes it possible to obtain all necessary data for analysis of complex systems without the need to determine their individual components.

How can one make a quantum system show the entire set of its quantum states? To do so, one can stimulate the object to go through the entire range of its possible transformations from its birth to, figuratively, end of its life. And if we make sure these transformations are cyclic, then it will certainly be possible to see a wide range of quantum states of the object manifesting themselves with a certain probability. There are two efficient ways to observe the set of quantum states of a solid in real time using point contacts. One of them is based on the manual control of the contact creation and destruction process using the “break junction” technology and multiple artificial repetition of the cycles in accordance with the forward-and-backward scheme^40^. The other way is absolutely natural and uses the possibilities provided by the cyclic switchover effect^41^. The cyclic switchover effect is an automatic repetition of dendrite growth and dissolution cycles which lead to the creation and destruction of dendrtic Yanson point contacts. This method can be realized thanks to the specific potential distribution in the Yanson point contact and emergence of an electrochemical gapless electrode system on the point-contact conduction channel when current flows through the contact immersed in a liquid medium.

Let us consider the needle-anvil electrode system, which is often used to create Yanson point contacts^37^, and place it in a liquid medium. The potential difference is applied to this system in the way that the positive polarity is observed at the anvil and negative polarity is observed at the needle. This gives rise to an ion current, which starts flowing through the electrolyte, and a dendrite begins to grow at the end of the needle, which serves as the cathode, towards the anvil. As soon as the tip of the dendrite reaches the opposite electrode, a dendritic Yanson point contact with direct conduction is formed there and a gapless electrode system appears on the conduction channel^32,41^. Due to the gapless electrode system, an anode space is formed on the conduction channel of the Yanson point contact on the anvil side and a cathode space is formed on the needle side. As a result, the dissolution process of the contact material in the area of the anode space begins, which ends with the complete dissolution of the dendritic conduction channel. At this point a cycle of nanostructural transformations in dendritic Yanson point contact ends, and a new cycle starts automatically without an external intervention, in which the stages of the described processes are repeated.

The uniqueness of the cyclic switchover effect is related to the fact that all transformation processes in dendritic Yanson point contact are controlled by the quantum shell effect. It is responsible for the crystalline structure of the contact formed as a result of the transitions from one metastable quantum state to another. Each metastable state of the Yanson point contact is characterized by a certain value of quantized electrical conductance^40,56^. Therefore, during the lifetime of a metastable state, the electrical conductance of the nanostructure remains unchanged. During the transition from one metastable crystalline state to another, the electrical conductance changes sharply by a certain number of conduction quanta^57,58^. After that, a rest period sets in again with a constant level of electrical conductance, which corresponds to the new metastable crystalline state of the dendritic Yanson point contact. These electrical conductance fluctuations lead to the appearance of steps in the dependence of the electrical resistance *R* of the system on time *t* ^41^. A step in the dependence *R(t)* characterizes a certain quantum state of the dendritic Yanson point contact, and a set of all steps shows all metastable quantum states of the dendritic point-contact system which are formed under certain conditions and can be interpreted as an integrated profile of the entire system^32^. In this case, as shown in Ref.^32^, a change in the environmental conditions, for example, addition of a gaseous agent, leads to a new set of quantum states of the dendritic point-contact system, which manifest themselves under the new specific conditions. Addition of a gas to the reaction volume changes the quantum profile of the entire system in a reproducible manner. The uniqueness and reproducibility of the quantum profile of the object under study can be demonstrated in the form of a conductance histogram. The conductance quantization of dendritic Yanson point contacts can serve as a new quantum mechanism for selective detection in gases and liquids.

The cyclic switchover effect and the quantum mechanism of selective detection in gases and liquids can be successfully used for detection in human breath. Registration of the spectral breath profile is carried out within the framework of the new quantum concept of detection in complex gaseous media in dynamic mode proposed in Ref.^32^. This approach allows it to be used both in a closed environment and in the ambient conditions.

**Figure 3:**
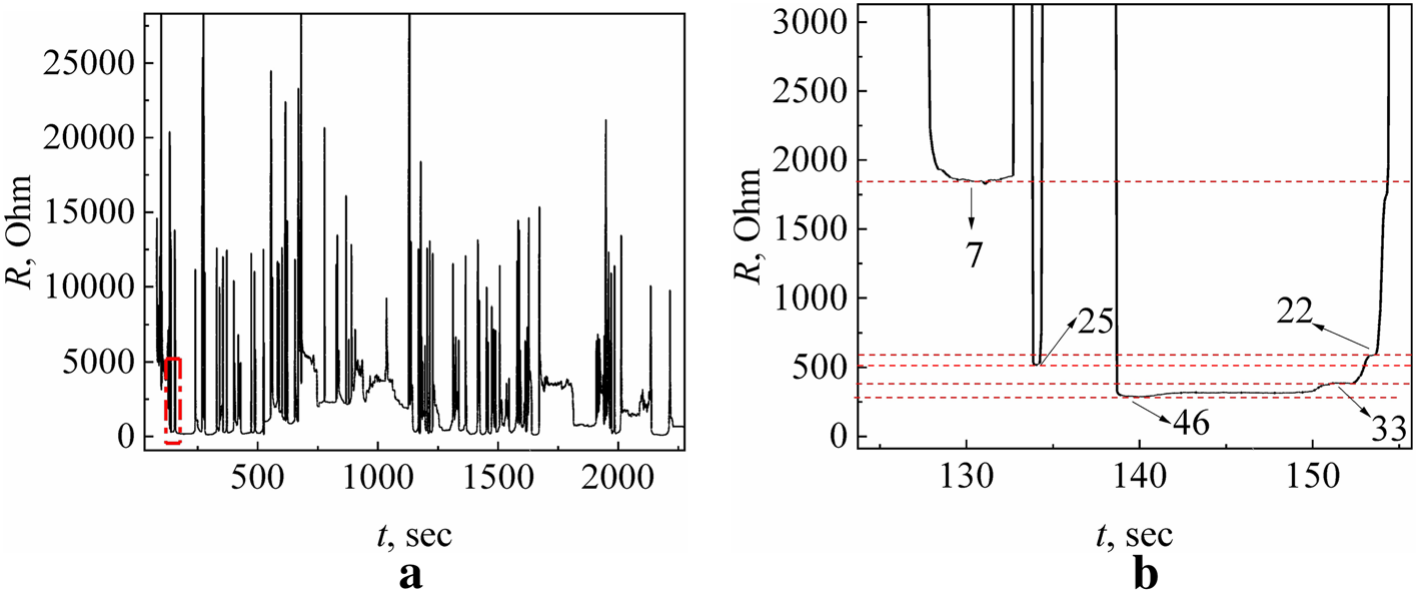
Electrical resistance variations of the quantum copper Yanson point contacts under action of human breath. (**a**) General view of *R*(*t*) dependence. (**b**) Enlarged section inside the red rectangle in (**a**). This section shows the set of metastable states of the quantum system in the environment of human breath. Arrows in (**b**) show the number of conductance quanta corresponding to a certain metastable state. *R* is the electrical resistance, *t* is the time.

To demonstrate the basic elements of the concept of detection in complex quantum systems in dynamic mode with the ultrahigh sensitivity of the quantum point-contact sensor operating in the environment, we carried out experiments with the following conditions. Dendritic Yanson point contacts were created using the "needle-anvil" technique in a cell to provide exposure to external atmospheric action. This technique is described in detail in Refs.^32,41,46^. The tip of the needle electrode was placed in a drop of pure (demineralized) water on the surface of the anvil and then moved towards the counterelectrode without letting them get into contact with each other. Then, an electric current was fed into the “needle-anvil” electrode system and the cyclic switchover effect was launched^41^. After several cycles of growth and dissolution of the dendritic conduction channel, the dendritic Yanson point contact was stabilized, and the process of its transformation was stopped for 30–60 s. During the stabilization period of the dendritic Yanson point contact, a volunteer breathed towards the quantum dendritic point-contact sensor located at a distance of 2–2.5 m from him/her, and the cyclic switchover effect automatically resumed 10–30 s later, which was a reaction of the quantum dendritic point-contact sensor to the action of human breath. The resistance changes of dendritic Yanson point contacts under the action of human breath were recorded in the dependence *R(t).* An example of the measured dependencies is shown in Fig. 3a. Here, the lower sections of the curve correspond to the contacts of a relatively large diameter created in the final phase of the synthesis process, while the higher sections correspond to the process of dissolution of the Yanson point-contact conduction channel resulting in the destruction of the contact, when the resistance of the system exceeds 12.9 kΩ.

**Figure 4:**
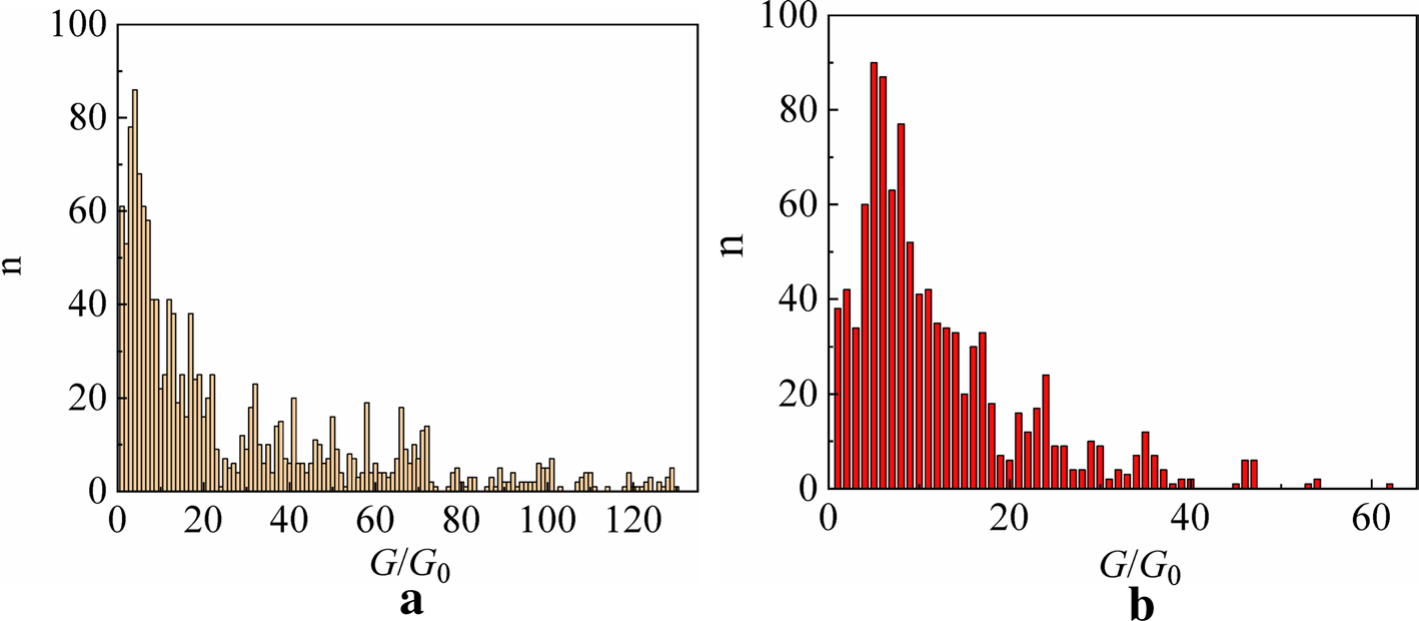
Conductance histograms of copper dendritic point contacts grown in the drop of pure water in the following environment: (**a**) breath; (**b**) ambient air. *G* is the conductance, *G*_0_ is the conductance quantum, *n* is the number of counts.

Figure 3b shows an enlarged section of the curve in Fig. 3a to demonstrate the presence of conductance steps corresponding to the metastable quantum states of the system. During the cyclic switchover effect, metastable states with a certain energy are repeated with a certain probability. As a result, a certain characteristic distribution of the system quantum metastable states is formed under the given conditions. In the considered case, the entire set of the quantum states of the system with their probabilities can be presented in the form of a conductance histogram (Fig. 4)^32,41^. The conductance histogram was plotted using the dependence of conductance *G* on time *t* obtained by an inverse transformation of the measured dependence *R(t)*.

The cyclic switchover effect makes it possible to record the energy profile of a human breath using dendritic Yanson point contacts in a reproducible manner. In the same way, those objects are used to find the distribution of quantum states in other quantum systems^32^. The conductance histogram of dendritic Yanson point contacts is a unique energy signature of a complex gas medium which provides the possibility of distinguishing one gas mixture from another. Figure 4 shows the human breath profile recorded in our experiment and the conductance histogram obtained in ambient atmosphere before the start of the breath experiment. The presence of a significant number of components in the human breath implies a large variety of states of the quantum system "dendritic Yanson point contact—human breath". This leads not only to a significant increase in the conductance histogram length, but also to a more complex histogram behaviour compared to a simpler mixture of atmospheric gases, which can clearly be seen in Fig. 4.

This demonstration is to show the unique capabilities of the quantum mechanisms for selective detection in complex gaseous media using quantum point-contact sensors operating within the framework of the dynamic mode concept. To apply this concept to the development of methods for medical diagnostics, it is more convenient to use specially designed devices^32,46^, in which, for the sake of purity and reliability of the data obtained, it is possible to isolate the studied environment from an external action. However, operation in ambient conditions also has great prospects for practical use. For example, remote detection of human breath may be of great interest for the development of detectors of human emotional states, which can be used in public spaces, such as airports, etc., to help prevent terrorist attacks^49^. As our preliminary studies have shown, a change in the human breath profile caused by a change in the emotional state cannot be hidden. A terrorist carrying out illegal actions, like any person, experiences emotional stress. This leads to a change in the metabolism of their body followed by a change in the composition and concentration of the components of their breath. This, in turn, leads to the changes in the spectral breath profile the person cannot prevent.

## Conclusions

The actively developing field of sensor research has great prospects for testing new quantum mechanisms for detection and analysis of complex systems and subsequent development of various innovative technologies. The search for such mechanisms is ensured by the knowledge of the nature of matter, which guarantees a comprehensive understanding of physical and chemical processes in complex systems at the atomic level and allows finding innovative approaches to their study and analysis. There is no doubt that in the near future one of the leading places among the approaches to solving the problems faced by sensor researchers will belong to the developments based on quantum processes and mechanisms.

The state of the problem and the ways to solve it can clearly be seen in the example of sensors with the operation principle based on electrical conductance of their sensing elements changing in response to the action of the analyzed agent. Their development, characterized by a gradual improvement in the parameters of the new devices and the absence of breakthrough solutions capable of providing a transition to a qualitatively new level, shows that the traditional approach to the development of sensors dealing with the macroscopic nature of substances is gradually exhausting itself. This is especially evident in the existing trends to increase the selectivity of the developed sensors and create devices for analysis of complex gas mixtures. Therefore, it becomes promising to attract new opportunities that can be provided by the transition from classical to quantum physics.

The operation principles of quantum sensors are based on the fundamental quantum mechanisms that ensure their functioning at a qualitatively different level compared to their classical counterparts. Their unique nature and the unlimited possibilities for a wide range of applications make quantum sensor mechanisms one of the leading candidates for solving complex sensor problems. For example, to develop quantum sensors, quantum processes involving energy transformation can be employed, which is virtually impossible to implement in conventional sensors and nanosensors. This makes it possible to use the energy approach as the basis of functioning of innovative quantum devices, which opens up unlimited possibilities for analysis of complex systems.

Since theoretical consideration reveals the advantages of using quantum mechanisms for detection in complex gas mixtures, it is necessary to create appropriate quantum tools for their practical implementation. One of such tools is the Yanson point contact and quantum point-contact sensors based on it. The quantum properties of point-contact sensors make it possible to record the spectral profiles of human breath which contain comprehensive information about this complex gaseous medium. Quantum point-contact sensors have already demonstrated their high efficiency, as well as the possibility of implementing various mechanisms and concepts of quantum analysis of breath. In particular, the implementation of the concept of characteristic parameters of the quantum sensor response signal made it possible for the first time to detect carcinogenic strains of the bacterium *Helicobacter pylori*^24^. Within this concept, a characteristic parameter of the spectral profile of human breath was determined, which allowed unambiguous differentiation between the carcinogenic and tolerant strains of the bacterium *Helicobacter pylori* in real time.

The wide possibilities of sensor analysis provided by the use of the spectral quantum mechanism for selective detection in complex gas mixtures were shown in the development of breath tests to determine human hormonal background^25,26,51^. The fundamental energy information about the quantum interactions in the "point-contact sensor—breath" system allowed the same problem to be solved by using different independent methods. As a result, it was shown that information about any substance in the human body which contributes to the breath can be obtained by both directly analyzing the spectral sensor profile of breath and using some characteristic parameters. This allows quick control over the obtained results, a widely available monitoring of the human hormonal state in real time, and provides a higher reliability of medical diagnostics with quantum point-contact sensors.

Conductance quantization as a new mechanism for selective detection in human breath can be an example which demonstrates the variety of quantum detection mechanisms and the unlimited possibilities for the development of new sensor technologies. The discovery of this mechanism^32^ entailed the creation of a new concept of quantum detection in complex gaseous media in dynamic mode. The development of this concept opens up new opportunities for the practical use of quantum point-contact sensors. This includes, in particular, remote detection in human breath. This approach is of great interest for the development of detectors of human emotional states, which can be used to thwart terrorist threats.

In this paper, we analyzed for the first time the nature of the new quantum mechanisms for selective detection in complex gaseous media which provide the highest possible efficiency of conductive quantum sensors. On the basis of these quantum mechanisms, the concepts of quantum detection and innovative methods of analysis are developed, which are virtually impossible to implement in conventional sensors and nanosensors. Examples of original solutions to problems in the field of detection and analysis of human breath using point-contact sensors have been considered. A new method of analysis based on detection of metastable states of the "point-contact sensor—breath" system in dynamic mode has been proposed. The conductance histogram of dendritic Yanson point contacts recorded for this system is a unique energy signature of breath which allows differentiation between the states of the human body. We demonstrate that nanoscale Yanson point contacts, which, thanks to their quantum properties, can replace a massive spectrometer, open up wide opportunities for solving complex sensor problems in the field of breath analysis using a new generation of portable high-tech quantum devices.

## Data Availability

Data has been provided with the manuscript. Additional information is available from the corresponding author upon reasonable request.
